# Platelet Rho GTPases–a focus on novel players, roles and relationships

**DOI:** 10.1042/BJ20141404

**Published:** 2015-03-06

**Authors:** Robert Goggs, Christopher M. Williams, Harry Mellor, Alastair W. Poole

**Affiliations:** *Department of Clinical Sciences, College of Veterinary Medicine, Cornell University, Ithaca, NY 14853, U.S.A.; †School of Physiology and Pharmacology, Faculty of Medical and Veterinary Sciences, University of Bristol, Bristol BS8 1TD, U.K.; ‡School of Biochemistry, Faculty of Medical and Veterinary Sciences, University of Bristol, Bristol BS8 1TD, U.K.

**Keywords:** cell division control protein 42 (Cdc42), Rif, RhoG, shape change, secretion, thrombosis, AP-MS, affinity-purification mass spectrometry, ARHGEF, Rho guanine nucleotide exchange factor, ARP2/3, actin related protein 2/3 complex, Cdc42, cell division control protein 42, CRP, collagen-related peptide, DOCK, dedicator of cytokinesis, ECM, extracellular matrix, ELMO, engulfment and cell motility, Ena, enabled, F-actin, filamentous actin, FcR, Fc receptor, GAP, GTPase activating protein, GDI, guanine nucleotide dissociation inhibitor, GEF, guanine nucleotide exchange factor, GP, glycoprotein, IMD, IRSp53 and MIM homology domain, ITAM, immunoreceptor tyrosine-based activation motif, LARG, leukaemia-associated Rho GEF (ARHGEF12), LAT, linker for activation of T-cells, MLC, myosin light chain, MTSS1, metastasis suppressor protein 1, NSF, *N*-ethylmaleimide-sensitive factor, PAK, P21-activated kinase, PAR, protease activated receptor, PF4, platelet factor-4, PI3K, phosphoinositide 3-kinase, PKC, protein kinase C, PLC, phospholipase C, PREX-1, PI(3,4,5)P3-dependent Rac exchanger 1 protein, Rac, Ras-related C3 botulinum toxin substrate, Rho GTPase, Ras homology family GTPase, Rif, Rho in filopodia (RhoF), ROCK, Rho kinase, SNARE, soluble *N*-ethylmaleimide attachment protein receptors, Syk, spleen tyrosine kinase, TCR, T-cell receptor, TRIO, triple functional domain containing protein, VAMP, vesicle-associated membrane protein, VASP, vasodilator-stimulated phosphoprotein, VWF, von Willebrand factor, WASP, Wiskott–Aldrich syndrome protein

## Abstract

Rho GTPases are critical for platelet function. Although the roles of RhoA, Rac and Cdc42 are characterized, platelets express other Rho GTPases, whose activities are less well understood. This review summarizes our understanding of the roles of platelet Rho GTPases and focuses particularly on the functions of Rif and RhoG. In human platelets, Rif interacts with cytoskeleton regulators including formins mDia1 and mDia3, whereas RhoG binds SNARE-complex proteins and cytoskeletal regulators ELMO and DOCK1. Knockout mouse studies suggest that Rif plays no critical functions in platelets, likely due to functional overlap with other Rho GTPases. In contrast, RhoG is essential for normal granule secretion downstream of the collagen receptor GPVI. The central defect in RhoG^−/−^ platelets is reduced dense granule secretion, which impedes integrin activation and aggregation and limits platelet recruitment to growing thrombi under shear, translating into reduced thrombus formation *in vivo*. Potential avenues for future work on Rho GTPases in platelets are also highlighted, including identification of the key regulator for platelet filopodia formation and investigation of the role of the many Rho GTPase regulators in platelet function in both health and disease.

## Abstract 2

In platelets, Rif interacts with cytoskeleton regulators including formins mDia1 and mDia3, whereas RhoG binds SNARE-complex proteins and cytoskeletal regulators ELMO and DOCK1. Knockout mice studies suggest RhoG is essential for dense granule secretion downstream of the collagen receptor GPVI.

## PLATELETS IN PHYSIOLOGY AND PATHOLOGY

The activities of Rho GTPase proteins are central to many of the processes that underpin the physiological and pathological roles of platelets [1]. Platelets are small, disc-shaped cells with a short lifespan that number in the hundreds of millions per millilitre of blood and are integral to the initial response to vascular endothelial damage [2,3].

*In vivo*, haemostasis is a complex continuum of events, initiated by damage or disruption of the normally continuous endothelial cell barrier that exposes subendothelial extracellular matrix (ECM). Platelets are activated by cell-surface receptor interactions with ECM ligands, leading to shape change, granule secretion, platelet aggregation and procoagulant surface expression. Locally generated thrombin, platelet-synthesized thromboxane and adenosine diphosphate (ADP) secreted from platelet dense granules stimulate platelets arriving at the site of injury, amplifying platelet responses to primary agonists and extending the platelet plug.

Activating interactions between platelets and von Willebrand factor (VWF) and collagen via the cell-surface receptors GpIb-IX-V and GPVI, respectively lead to dramatic alterations in shape, due principally to reorganization of the actin cytoskeleton. Alterations in platelet morphology increase surface area, maximizing opportunities for interactions with ECM and other cells [4,5]. These initial shape change events are loss of the discoid shape, sphering and then extension of filopodia [6]. These thin (0.1–0.3 μm) finger-like membrane protrusions enclosing tight parallel bundles of filamentous actin (F-actin) increase the potential for contact between platelets and extracellular matrix and for interactions with other cells [7]. Later, cell shape changes involve lamellipodia formation to allow platelet spreading [8], enabling platelets to cover endothelial surface defects.

Granule secretion is critical for normal platelet function and is a highly orchestrated and regulated process. In platelets, some granules may be pre-docked at the plasma membrane, but most are homogeneously distributed through the platelet cytosol and must be brought to the plasma membrane or membranes of the open canalicular system for release to occur [9,10]. Granule secretion requires reorganization of the actin cytoskeleton to facilitate granule transport and enable granule access to platelet membranes. These cytoskeletal rearrangements occur prior to the soluble N-ethylmaleimide-sensitive factor (NSF) attachment protein receptor (SNARE) machinery-mediated regulation of the membrane fusion process. The Rab family of small GTPases are involved in platelet granule formation and Rabs 3b, 6, 8 and 27a are all involved in activation-triggered granule secretion [11–14]. The differential roles of individual Rab proteins in regulation of distinct platelet and megakaryocyte secretion events are still being clarified [15], but a thorough discussion of platelet Rab proteins is outside the scope of this review.

Mammalian platelets are highly specialized for haemostasis but also contribute to antimicrobial host defence through expression of cell-surface receptors for immunoglobulins [16], pathogen-associated molecular patterns [17], and chemokines [18]. Platelets can directly bind bacteria through multiple receptor–ligand interactions that trigger platelet shape change [19], characterized by filopodia formation with coincident rearrangement of both the platelet actin cytoskeleton and microtubule networks [20–22]. Platelets also secrete antimicrobial proteins including thymosin β-4, platelet kinocidins and complement proteins from their α granules [23], which can be released in direct response to interactions with bacteria [24,25]. Haemostatic accumulations of platelets at vascular injury sites also place them in the ideal location to promote and co-ordinate revascularization and tissue repair through secretion of angiogenesis activators, growth factors and chemokines from their α granules [26,27].

Platelets are implicated in pathological processes typically due to inappropriate or excessive activation of normal platelet physiological processes. The best-known example is atherothrombosis, where collagen exposure and local thrombin generation at sites of plaque rupture leads to exuberant thrombus formation and consequent ischaemia or infarction, most commonly occurring in coronary and cerebral arteries. Platelets may also be involved in the pathogenesis of atherosclerosis itself [28]. At predilection sites for atherosclerosis and in the presence of risk factors such as hypercholesterolaemia, platelets may adhere to intact endothelium and stimulate endothelial cells to recruit leucocytes that become the foam cells characteristic of atherosclerosis [29–31]. Platelets also play complex roles in the development and resolution of such varied diseases as diabetes [32], sepsis [33] and cancer [34].

## Rho GTPases

**Figure 1:**
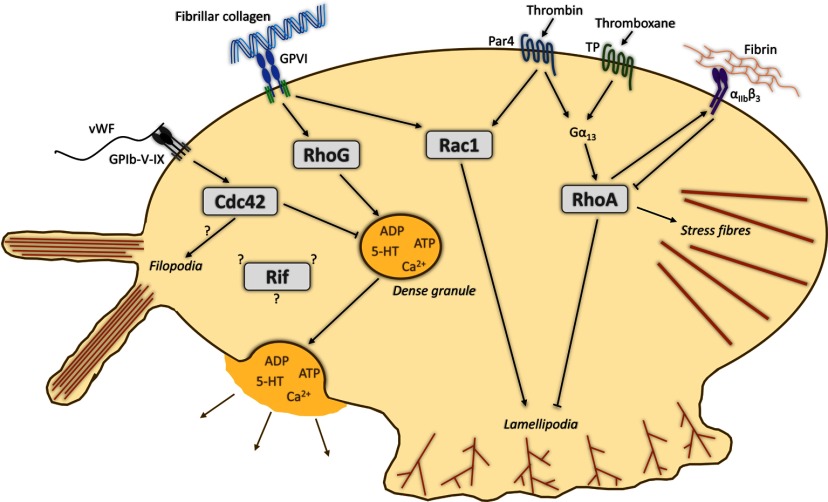
A schematic representation of the known roles of Cdc42, Rac1 and RhoA in the shape change and granule secretion functions of platelets The potential roles of RhoG and Rif are also included for comparison. Cdc42 plays a role in regulating dense granule secretion and may be partly responsible for platelet filopodia formation. Rac is involved in lamellipodia formation downstream of both collagen-GPVI and thrombin interactions with PARs. RhoA is also involved in regulating lamellipodia formation, integrin activation and is responsible for generating stress fibres.

Platelets contain classical Rho family members from all four subfamilies and also atypical Rho GTPases such as RhoBTB1 [35]. The Rho GTPases Rac [8], RhoA [36] and Cdc42 [37], have established roles as regulators of platelet function (Figure 1). Transcript and proteomic studies suggest human platelets also express other classical Rho family members including RhoB, RhoC, RhoH and RhoQ but the functions of many of these other GTPases in platelets are unknown [35,38]. One major function of Rho GTPases is regulation of the actin cytoskeleton, but they are also involved in a number of other biochemical pathways. Of most relevance to platelets are control of phospholipase C (PLC) [39], and phosphoinositide-3-kinase (PI3K) [40], and the regulation of microtubule dynamics [41], cell–cell contacts [42], and granule secretion [43]. This functional diversity is accomplished through the binding of a wide range of effector proteins to the GTP-loaded activated GTPase. These effectors belong to multiple protein families including actin nucleation proteins such as the diaphanous related formins and the ARP2/3 complex, actin binding and bundling proteins such as enabled/vasodilator-stimulated phosphoprotein (Ena/VASP) and membrane deforming proteins such as the IRSp53 and MIM homology domain (IMD) containing protein MTSS1 (metastasis suppressor protein 1) (Table 1) [1]. Although there is considerable variation in the motifs that different effectors use to bind Rho GTPases, a number of effectors share consensus binding regions such as the Cdc42/Rac-interactive-binding (CRIB) domain found in Wiskott–Aldrich syndrome protein (WASP) and P21-activated kinase (PAK). Among the Rho GTPase effector proteins of particular importance to actin cytoskeletal rearrangements in platelets are proteins of the WASP/WAVE/Scar family and the diaphanous-related formin proteins [44,45].

**Table 1:** Platelet Rho GTPase effectors A list of known effector proteins for RhoA, Rac1 and Cdc42 compiled from Bishop and Hall [113] and Bustelo et al. [144] and cross-referenced against mRNA transcripts from human platelets [35], and two recent proteomic databases such that only proteins known to be expressed are listed [145,146].

Effector	Protein type (alternate protein name)	Upstream GTPase	Main biological function
FilaminA	Actin binding protein	RhoA, Rac1, Cdc42	Cytoskeletal regulation, actin filament cross-linking
CopG2	Coatomer protein (γ2-Cop)	Cdc42	Vesicle trafficking (clathrin route)
DIAPH1,2	Formin	RhoA, Rac1	Cytoskeletal change via profilin and IRSp53
FHOD1	Formin	Rac1	Cytoskeletal and transcriptional regulation
FMNL1	Formin	Rac1	Cytoskeletal organization, cell polarity, cytokinesis
IP3R1	Inositol 1,4,5-triphosphate receptor	RhoA	Calcium entry in endothelial cells
PI4,5PK	Lipid kinase	RhoA	Phosphatidylinositol bisphosphate level modulation
CybA	NADPH oxidase complex subunit	Rac1	Superoxide production
NCF1,2	NADPH oxidase complex subunit	Rac1, Cdc42	Superoxide production
PLC-β2	Phospholipase, C type (PLC-β2)	Cdc42, Rac1	Production of second messengers
KCNA2	Potassium Channel subunit	RhoA	Potassium entry
PIK3R1	Regulatory p85 subunit of PIK3C	Rac1, Cdc42	Regulation of PIK3C activity, signal transduction
PPP1R12A	Regulatory subunit of phosphatase1	RhoA	MLC inactivation, cytoskeletal regulation
IQGAP1,2	RhoGAP and scaffold protein	Rac1, Cdc42	Cytoskeletal regulation, cell–cell contacts
ARFIP2	Scaffold protein (Por1)	Rac1	Cytoskeletal regulation
Cdc42SE1,2	Scaffold protein (Spec1,2)	Rac1, Cdc42	Modulation of GTPase signalling outputs
CYFIP1,2	Scaffold protein (Pir121)	Rac1	Regulation of the cytoskeleton via WASF proteins
MTSS1	Scaffold protein	Rac1	Cytoskeletal organization via WASF/WAVE
Kinectin1	Scaffold protein	RhoA, Rac1, Cdc42	Kinesin binding, microtubule vesicular trafficking
NCK1	Scaffold protein with SH2/3 domains	Rac1	Complex formation with WASP, signal transduction
NCKAP1	Scaffold protein (Nap125, Nap1)	Rac1	Regulation of the cytoskeleton via WASF proteins
N-WASP	Scaffold protein	Cdc42	Cytoskeletal regulation via Arp2/3 complex
Pard6 A,G	Scaffold protein (Par6α,γ)	Rac1, Cdc42	Cell polarity. Links GTPases and atypical PKCs
Trip10	Scaffold protein	Cdc42	Binding of WASP to microtubules
WASP	Scaffold protein	Rac1	Cytoskeletal regulation via the Arp2/3 complex
WAVE/Scar1,2	Scaffold protein	Cdc42, Rac1	Cytoskeletal regulation via the Arp2/3 complex
Cdc42bpgB	Serine/threonine kinase (MRCKβ)	Rac1, Cdc42	Cytoskeletal regulation
p70S6K	Serine/threonine kinase	Cdc42	Regulation of translation, cell cycle
PAK2	Serine/threonine kinase	Rac1, Cdc42	Cytoskeletal organization, kinase activation
PKCα	Serine/threonine kinase	RhoA, Rac1, Cdc42	Signal transduction
PKN1,2	Serine/threonine kinase (Prk)	RhoA	Vesicle recycling, PLD1 activation
ROCK1,2	Serine/threonine kinase (Rok α,β)	RhoA	Cytoskeleton, blockage of cell contact inhibition
Stat3	Transcriptional factor	Rac1, Cdc42	Transcription
α-Tubulin-1C	Tubulin	Rac1	Integral component of microtubules

## RhoA

In platelets, RhoA activation causes the initial platelet sphering seen after activation [46]. Following stimulation of platelets with thrombin or the thromboxane mimetic U46619, RhoA is activated by ARHGEF1 (p115RhoGEF) [47]. This RhoA GEF is stimulated by activity of G_α13_ subunits of the thrombin and thromboxane receptors [46,48,49]. RhoA may act through mDia1 to nucleate new actin filaments and through ROCK and LIM-kinase to stabilize actin filaments. RhoA is also able to cause myosin contraction through myosin phosphatase targeting protein phosphorylation and hence inhibition of myosin light chain (MLC) phosphatase activity [50].

RhoA regulation in platelets is complex, because after initial activation, signalling from αIIbβ3 inhibits the GTPase allowing spreading to occur [51]. Subsequently, this inhibition is released and RhoA is then able to help stabilize thrombi under shear [52], and to mediate stress fibre formation and clot retraction [53]. In fibroblast cell cultures, RhoA expression promotes actin stress fibre formation and focal adhesions [54]. Several RhoA effector proteins have been implicated in the formation of these structures, including ROCK and mDia1 [55,56]. Stress fibre formation by thrombin-stimulated platelets also involves the interaction of RhoA with mDia1 in a PI3K-dependent manner (Figure 1) [57]. Studies of platelets lacking RhoA have confirmed the data obtained by pharmacological means and show that RhoA is required for integrin activation, granule secretion and clot retraction and that these defects translate into haemostatic and thrombotic defects *in vivo* [36].

## Cdc42

Cdc42 is an established regulator of filopodia formation. The prototypic pathway for filopodia formation involving Cdc42-WASP-ARP2/3 was based on studies of fibroblasts [58], but other Cdc42 driven pathways to filopodia also exist. Cdc42 binds the formins mDia2 and mDia3 [59,60], and can activate IRSp53, which binds mDia1 [61,62]. Cdc42 also promotes filopodia formation through the Ena/VASP protein Mena [63]. Experiments in fibroblasts show Cdc42 is not absolutely required for filopodia formation, however [64], and additional novel Cdc42-independent pathways have been identified involving the Rho GTPase Rif [65], and lipid-phosphatase-related protein-1 (LPR1) [66].

Cdc42 is highly expressed in platelets (~28000 copies) [38], and is activated by thrombin stimulation or when platelets spread on collagen [67,68]. Cdc42 translocates to the actin cytoskeleton upon stimulation of protease activated receptors (PARs) or P2Y receptors, an event that requires integrin activation, actin polymerization and tyrosine kinase activity [69,70]. Although it is known to be activated in platelets, the extent of Cdc42's functions in platelets is presently unclear. Studies using the GTPase inhibitor secramine A suggested that Cdc42 was required for platelet filopodia formation [71,72]. The selectivity of this inhibitor has been questioned, however [37], and two studies have since used distinct mouse gene deletion strategies to study the function of Cdc42 in platelets.

Use of a PF4-Cre driven system to produce megakaryocyte- and platelet-specific deletion of the Cdc42 gene in mice resulted in mild macrothrombocytopenia (~500×10^3^/μl) and shortened platelet lifespans [37]. Critically and surprisingly, Cdc42^−/−^ platelets formed filopodia with normal morphology on fibrinogen-coated surfaces [37], demonstrating that Cdc42-independent pathways to filopodia formation exist in platelets. That study found that Cdc42 was not fully redundant in platelets, however, because filopodia formation on VWF was defective in Cdc42^−/−^ platelets suggesting Cdc42 may couple specifically to GPIb. The principal phenotype of platelets from these mice was increased secretion in response to GPVI agonists, thrombin and the thromboxane mimetic U46619, suggesting that Cdc42 normally functions as a negative regulator of platelet secretion. This augmented secretion translated into increased thrombus formation *in vivo*, but oddly these mice had increased tail-bleeding times.

A subsequent comparable study used an Mx-Cre gene deletion system to induce excision of the Cdc42 gene in haematopoietic cells by administration of polyinosinic acid–polycytidylic acid. The phenotype of these mice is more consistent with the hypothesized functions of Cdc42 in platelet function including impaired filopodia formation and reduced spreading, secretion and aggregation responses [73]. The discrepancies between these studies may well relate to methodological differences, but leave unanswered questions about platelet filopodia formation and have fed debate in the field about the best strategy for generation of platelet-specific conditional knockout mice [74]. It is clear from the PF4-Cre mice data, however, that even when platelet Cdc42 protein expression is ablated, these platelets still retain certain functions including filopodia formation and secretion. As such, this finding suggests that other pathways to these processes exist in platelets.

## Rif

The small GTPase Rif (RhoF) was identified from partial cDNA sequences during a search for novel Rho-family GTPases and displays relatively low homology with other Rho proteins, with the exception of RhoD [75]. In contrast with Rac1, RhoA and Cdc42, Rif is expressed in only a subset of tissues [76]. Messenger RNA for Rif has been identified in human megakaryocytes [77], and in human and mouse platelets [35].

Our understanding of endogenous Rif is limited, although it is expressed at higher levels in malignant lymphoma cells, compared with normal B-lymphocytes, suggesting Rif overexpression promotes malignant transformation [78]. Using recombinant Rho protein affinity purification-mass spectrometry (AP-MS) our group has identified interacting protein networks for Rif in platelets and confirmed some of these interactions by immunoblotting (Goggs, Mellor and Poole, unpublished observations). Although the interaction of Rif with the formins has been documented in other cell types, our work suggests Rif is able to interact with mDia1 and mDia3 in platelets, which hints at its function.

Ectopic Rif expression in cultured cells promotes formation of long, thin, flexible, actin-rich protrusions [79,80]. In contrast, the filopodia formed by Cdc42 are shorter, thicker and emerge only from the cell periphery [81]. It is possible that filopodia induced by Cdc42 and those induced by Rif represent different subtypes. Alternatively, generation of distinct structures may result solely from different molecular mechanisms of formation, since Rif directly binds and activates both mDia1 and mDia2, whereas Cdc42 does not directly activate mDia1 [59,61,65]. Rif also generates filopodia through interactions with the formin proteins mDia1 and mDia2 in various cell types in culture [61,65], and can induce stress fibre formation in HeLa cells [82]. Once activated by Rif, the formin proteins directly nucleate actin filament polymerization at filopodia tips [65,83], and as such provide a pathway to filopodia formation that is independent of Cdc42. Both filopodia and stress fibres are important for platelet function, enhancing cell–cell and cell–matrix interactions and facilitating clot retraction respectively. Previous studies using platelets from gene deletion mice suggest that the canonical pathway to filopodia formation is not the only means by which filopodia form in platelets [25,26,37], and functional overlap between Cdc42 and Rif is probable on the basis of shared interactions with mDia2 [84].

Our group recently generated a novel constitutive Rif knockout mouse line to test the hypothesis that Rif might provide an alternative, Cdc42-independent route to filopodia formation and to evaluate the role of Rif in platelets [85]. The mice were viable and showed no overt phenotype. Evaluations of multiple aspects of platelet function including assessments of inside-out and outside-in signalling through integrin αIIbβ3 and secretion from α and dense granules did not identify an essential role for Rif in mouse platelets, however. In addition our data suggest that Rif is not required for the actin rearrangements in megakaryocytes that facilitate thrombopoiesis, since platelet counts were comparable with wild-type controls [85]. Most importantly, static adhesion and flow chamber assays suggested that Rif is not essential for platelet filopodia formation or for manipulating the actin cytoskeleton during other platelet shape change events.

Although the lack of an identified role for Rif in mouse platelets does not preclude a role for the protein in human platelets, the question of which Rho GTPase(s) regulate platelet filopodia formation remains open. The most probable explanation for the lack of an observed phenotype in the Rif^−/−^ mice, is that Rif plays redundant roles in platelets for which other Rho GTPases can substitute. Phylogenetic analysis of Rho GTPases suggests that the functions of Rif are most likely to overlap with RhoD [58], but analysis of platelet mRNA sequences and proteomics data suggests that RhoD is not expressed [35,38]. Interestingly, a recent study of platelets in the mDia1^−/−^ mouse found no defects in clot retraction or in fibrinogen binding, P-selectin surface expression, or cell spreading in response to either collagen-related peptide (CRP) or thrombin stimulation [86]. As such, data from the mDia1^−/−^ mouse is consistent with analyses of Rif^−/−^ platelets and suggests pathways other than Rif-mDia1 regulate filopodia formation in platelets. Functional overlap with Cdc42 seems most likely given the common binding partners and data which suggests Cdc42 and Rif co-operate during development of dendritic spines from dendritic filopodia [87].

## Rac

Although three Rac isoforms exist, only Rac1 and Rac2 are expressed in platelets [38]. Rac1 activation occurs downstream of collagen–GPVI interactions and thrombin activation of PARs [39,88]. Platelet adhesion through integrin α_2_β_1_ initiates outside-in signalling and activation of tyrosine kinases and small GTPases, including Rac1, which is involved in cross-talk between integrins in collagen-adherent platelets [89]. Studies using platelets from Rac1/Rac2 double null mice suggest that Rac promotes lamellipodia formation but not filopodia formation [8]. The Rac1-dependent formation of lamellipodia in platelets likely occurs via WAVE/Scar activation of the ARP2/3 complex [90]. In other cells, Rac acts in tandem with the protein gelsolin [91], generating novel barbed ends from which the Rac-activated ARP2/3 complex can nucleate new branched filaments at the lamellipodia leading edge (Figure 1) [92]. Such links are plausible for platelets also, but have not been explored to date. Rac1 is essential for GPVI-regulated platelet spreading and for sustaining shear-resistant platelet aggregates under flow both *in vitro* and *in vivo* and may also be involved in granule secretion [43].

Data from a recent study of conditional Rac1/Cdc42 double null mice suggest that additional understanding of the overlapping roles of related proteins can emerge from such studies [93]. The platelet/megakaryocyte selective Rac1/Cdc42^−/−^ mice had marked macrothrombocytopenia, abnormal platelet ultrastructure characterized by irregular granule distribution and impaired platelet function. Some of these abnormalities overlapped with those described for platelets from mice with single gene deletions, for instance an inability of Rac1/Cdc42^−/−^ platelets to spread on fibrinogen. The present study again used the PF4-Cre system for gene deletion and demonstrated that Rac1/Cdc42^−/−^ platelets are still able to form filopodia. Some novel abnormalities were identified in the double gene deletion mice, particularly the abolition of proplatelet formation, a phenotype that was associated with defective tubulin organization, whereas actin assembly and structure were largely preserved.

## RhoG

RhoG is a classically regulated GTPase, most closely related to Rac [94]. Several GEFs and guanine nucleotide dissociation inhibitors (GDIs) for RhoG have now been identified [95–98], as well as a number of key effector proteins. In various cell types, RhoG regulates the actin cytoskeleton and is involved in filopodia formation [99], membrane ruffling [100], neurite outgrowth [101,102], T-cell spreading [103], dendritic spine morphogenesis [104] and lamellipodia formation [105]. The pathways to these various structures involve diverse effector proteins and may be separately regulated by GEFs such as Vav [106], and triple functional domain containing protein (TRIO) [100,107]. Unusually, however, the involvement of RhoG in these processes is frequently through upstream regulation of Rac1 and/or Cdc42.

The regulation of Rac1 by RhoG likely occurs through recruitment of engulfment and cell motility (ELMO) family proteins [105,108], and the dedicator of cytokinesis (DOCK) family of Rac1 GEFs [104,109]. The complex of RhoG–ELMO–DOCK1 proteins is required for integrin-mediated cell spreading and neurite outgrowth in PC12 cells [108]. This work has since been extended to demonstrate that Rac activation through ELMO and DOCK proteins enables RhoG to control lamellipodia formation in epithelia [110], spine morphogenesis in hippocampal neurons [104], and front–rear polarity and cell migration in keratinocytes [105]. The functions of RhoG in lymphocytes have been investigated in cell culture systems [103], and using constitutive RhoG^−/−^ mice [111]. In B- and T-lymphocyte cell culture systems, RhoG is activated by Vav-1 and enhances gene transcription, particularly following ligation of the T-cell receptor (TCR) [103].

It is apparent that many of the activities driven by RhoG in other cells correspond closely with key platelet functions. Furthermore, the potential for RhoG to control Rac and/or Cdc42 in platelets is clearly of interest given the known importance of Rac and Cdc42 to platelet function. The equivalent receptors in platelets to lymphocyte TCRs are FcγRIIa–a low-affinity immunoglobulin receptor (not present in mouse platelets)–and the immunoreceptor tyrosine-based activation motif (ITAM) domain containing GPVI–FcRγ complex, which is the principal platelet receptor for collagen. By extension, this suggests that in platelets, RhoG may be involved in the signalling pathways downstream of GPVI.

Potential RhoG effector proteins in platelets have recently been identified using GST–RhoG AP-MS assays [112]. Proteins that specifically bound to active RhoG were identified and grouped by function and the interaction of selected candidate proteins confirmed by immunoblotting. Several plausible interactions were identified, including with ELMO and DOCK1 (DOCK180) and with regulators of the actin cytoskeleton and granule secretion machinery components such as VAMP2. These interactions have clear implications for the potential roles of RhoG in platelet function in both haemostasis and thrombosis. The action of ELMO is to enhance the GEF activity of DOCK1 for Rac. Thus, RhoG may activate Rac and hence manipulate the actin cytoskeleton or control cellular processes via protein and lipid kinases [113]. RhoG also interacts with several other DOCK proteins (DOCK5, DOCK10) expressed in platelets [114]. These proteins are also GEFs for other Rho GTPases, suggesting that RhoG may act as a hub controlling activation of several signalling pathways.

Two groups, including our laboratory recently independently investigated the role of RhoG in platelets using the same RhoG^−/−^ mouse line [112,115]. Both studies demonstrated that integrin activation, aggregation, α and dense granule secretion in response to GPVI agonists are significantly decreased in RhoG^−/−^ platelets, whereas responses to agonists of the PARs are normal. This reduced secretion in the absence of RhoG diminished positive autocrine and paracrine feedback and hence reduced platelet activation. Secretion defects in the absence of RhoG led to reduced formation of arterial thrombi, demonstrated using two different thrombosis models. The contribution of RhoG to dense granule secretion is particularly important in this context. Platelet ADP secretion is necessary for stabilizing thrombi under shear conditions and co-stimulation with ADP ameliorated the integrin activation and aggregation defects in the RhoG^−/−^ platelets. One study suggested that RhoG activation by GPVI and the hemi-ITAM domain containing receptor CLEC-2 are dissimilar since aggregation in response to the CLEC-2 agonist, fucoidan, was normal [115]. This may suggest that the FcRγ chains (associated with GPVI but not with CLEC-2) may be involved in RhoG signal transduction.

The thrombotic defects in RhoG^−/−^ mice were not paralleled by a haemostatic abnormality, however, since RhoG^−/−^ mice tail bleeding times were normal and there was no evidence of a bleeding propensity. It is possible that intact PAR-mediated signalling in RhoG^−/−^ mice was sufficient to compensate for the abnormal granule secretion after collagen stimulation, or that the tail bleeding assay employed was insensitive to collagen pathway defects [116].

The defect noted in RhoG^−/−^ platelets downstream of GPVI but not the PARs is most likely explained by the divergent signalling pathways engaged by these receptors. Stimulation of GPVI activates Syk (spleen tyrosine kinase), leading to the assembly of the linker for activation of T-cells (LAT) signalsome, consisting of various protein and lipid kinases and adapter proteins. Syk activates PLCγ2, which then activates PKC and increases intracellular calcium. In contrast, PARs couple to G_αq_ and G_α13_. Signalling through G_α13_ activates RhoA and hence ROCK, whereas G_αq_ signals through PLCβ to activate PKC and increase intracellular calcium. Both pathways activate RhoG, but it seems likely that RhoG activation is integral to the GPVI pathway to granule secretion, whereas RhoG activation following thrombin stimulation may be a secondary event.

Regulation of granule secretion by Rho GTPases can occur through actin cytoskeletal manipulation, granule biogenesis and intracellular calcium signalling regulation [37,39,117], but none of these were responsible for the phenotype in RhoG^−/−^. Direct links between active RhoG and regulators of these granule secretion events, including interactions with regulators of the actin cytoskeleton and the microtubule network, both of which facilitate platelet granule release, might provide an explanation [112,118]. Direct interactions between RhoG, SNARE proteins and SNARE regulators represent an important avenue for future study since similar connections have been described for Cdc42 and syntaxin [119].

### RhoG regulation and relationships

The specific interaction of RhoG with ELMO and DOCK1 in human platelet lysates, raises the possibility that RhoG might activate Rac in platelets. Investigations of this possibility using G-LISA assays, cofilin phosphorylation and platelet spreading did not suggest a defect in Rac activation in the absence of RhoG however [112]. This does not preclude Rac activation by RhoG through DOCK and ELMO in platelets, but it does show that Rac activation downstream of GPVI can occur via RhoG independent routes, potentially through the GEF activity of Vav.

The GEF for RhoG in platelets is unknown. The LAT signalsome that assembles after GPVI activation includes the GEFs Vav1 and Vav3. In fibroblasts and in T-lymphocytes, Vav proteins act as RhoG GEFs [111,120]. Interestingly, Vav1/Vav3^−/−^ mice have a similar phenotype to the RhoG^−/−^ mice including reduced platelet aggregation responses to CRP, but normal aggregation responses to thrombin. Similarly, Vav1/Vav3^−/−^ platelets also adhere poorly to collagen under shear. In contrast with RhoG^−/−^ platelets, PLCγ2 phosphorylation is reduced in response to CRP in Vav1/Vav3^−/−^ mice. This suggests that Vav is involved in PLCγ2 phosphorylation and that RhoG lies downstream of this event, consistent with RhoG activation being dependent on Vav when platelets are stimulated with CRP.

Although Vav1 is phosphorylated in platelets by thrombin stimulation [121], the phenotype of Vav1/Vav3^−/−^ mice suggests Vav proteins do not mediate platelet function following PAR activation. In turn, this may explain why RhoG is not required following thrombin stimulation of platelets. The finding that PLCγ2 phosphorylation is unaffected by RhoG expression suggests that two signalling pathways may follow LAT signalsome assembly: one mediated by PLCγ2 and an alternative route linking RhoG to granule secretion independent of intracellular calcium or PKC, perhaps via direct interactions with SNAREs.

A possible alternative to Vav is TRIO, a Rho-GEF that can mediate activation of Rac1, RhoG and RhoA [122]. TRIO is expressed at the transcript and protein levels in platelets, although there are no reports of its function to date [35,38]. Recently, it has been demonstrated in neuroblastoma cells that TRIO is tyrosine phosphorylated by Fyn and that this phosphorylation enhances Rac activation [123]. It is therefore plausible that Fyn phosphorylation of TRIO might accelerate RhoG activation downstream of GPVI in platelets.

**Figure 2:**
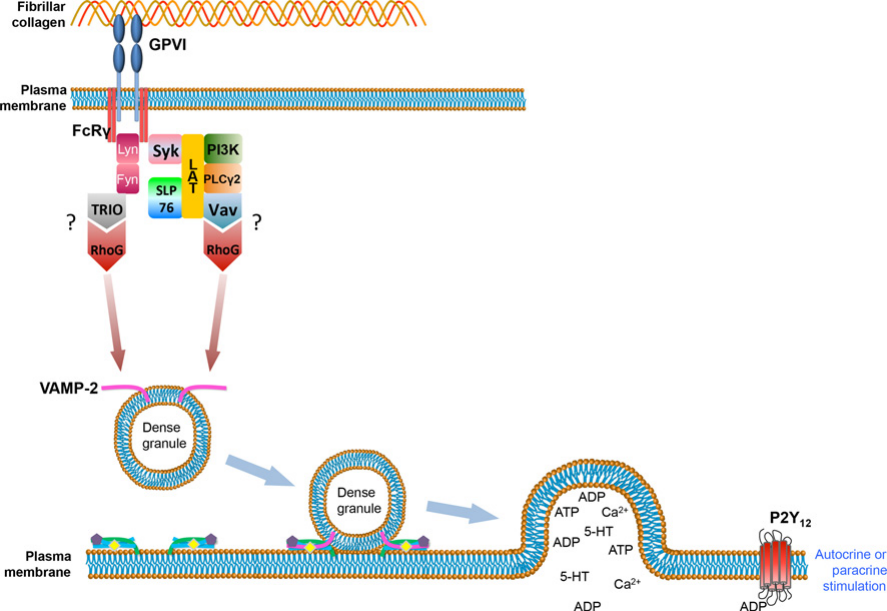
A schematic overview of the role of RhoG in the regulation of platelet secretion The interaction of fibrillar collagen with GPVI leads to the dimerization of the receptor and the association of the ITAM domains of the FcRγ chains. These are phosphorylated by Src family kinases Lyn and Fyn. In the model suggested by the Kunapuli group data, Fyn phosphorylates the Rho GEF TRIO, which in turn activates RhoG [115]. In the model supported by data from our laboratory [112], the activity of the Src family kinases leads to the activation of Syk [112]. The activity of Syk then assembles various signalling adapters, kinases and GEF proteins including Vav, which leads to the GTP loading of RhoG. Active RhoG then interacts with SNARE regulators and SNARE proteins including VAMP2 to promote dense granule secretion. Autocrine and paracrine feedback signalling then occurs through P2Y_12_ receptors.

Potentially significant differences exist in the recent analyses [112,115] of RhoG^−/−^ platelets, relating to the role of RhoG in Syk phosphorylation and to the point of activation of RhoG downstream of GPVI (Figure 2). One set of data [112] suggests RhoG is activated downstream of Syk and that RhoG is not required for Syk phosphorylation. In contrast, the other study [115] found that CRP-induced Syk phosphorylation was reduced in RhoG^−/−^ platelets and that RhoG activation was independent of Syk activity. That would suggest RhoG activation occurs prior to Syk activation, which would support a role for TRIO–phosphorylated by Fyn–as the GEF for RhoG. It may be that differences in platelet preparations, assay conditions or timings are responsible for these discrepancies in signal pathway analysis. Identification of the GEFs for RhoG acting downstream of GPVI in platelets may help to clarify this issue.

## PROSPECTS FOR FUTURE INVESTIGATION

Perhaps because platelets rely on Rho GTPases to orchestrate many of their critical functions, several early insights into the function of Rho GTPases were gained from studies of platelets [1], and they remain an excellent model system for the investigation of Rho GTPase function. Although the anucleate nature of platelets does preclude certain genetic manipulation, such disadvantages are countered by the ease with which millions of platelets can be acquired for study. Furthermore, the availability of multiple *in vitro* and *in vivo* assays of platelet function enables the roles of Rho GTPases to be dissected. There remain a number of Rho GTPases in platelets including RhoH, RhoJ and RhoBTB that are yet to be characterized, and a number of as yet unanswered questions, discussed below, suggesting there is considerable scope for further investigation in this field.

### What is the function of Rif and how do platelets form filopodia?

Our recent investigation of the function of Rif in platelets concluded that Rif is apparently dispensable for platelet function because it has redundant roles with other GTPases, although studies of megakaryocytes in Rif^−/−^ mice might provide additional information about the function of Rif in this cell lineage. Additional insight into the functions of Rif might be gained from looking at the functions of its effector proteins, since some are novel and uncharacterized. Additional cell culture work confirming and clarifying the nature of these interactions and identifying the probable functions of these effectors could be undertaken prior to returning to *in vivo* systems.

In order to test whether Cdc42 is compensating for Rif in the Rif^−/−^ mice, strategies to inhibit or prevent the function of Cdc42 could be employed. Pharmacological inhibition of Cdc42 with compounds such as secramine A [124], has previously been used to investigate the role of Cdc42 in platelets [125]. Since the selectivity of secramine A has been questioned, the preferable alternative would be genetic manipulation to produce mouse platelets devoid of both Rif and Cdc42. This is no small undertaking, however, since Cdc42^−/−^ platelets can only be produced through conditional targeting. Although challenging to perform, such follow-up work may be necessary to identify the critical regulators of filopodia formation by platelets.

The functional importance of filopodia to platelets suggests continued investigation of the mechanics of their formation is warranted. Various candidate proteins have been investigated but few critical proteins have been identified. A systematic, methodical approach investigating both Rho GTPases and their effector proteins will likely be necessary in order to identify the key regulators. It was reported in 2002 that ARP2/3 activity is essential for platelet filopodia formation [126], but this has not been replicated to date. This is of particular interest in the light of evidence that Cdc42 may be dispensable for platelet filopodia formation and given the potential for interactions between Rif and ARP2/3 complex components to occur in platelets. The lack of previous studies related to the ARP2/3 complex in platelets may relate to the lack of viable knockout mice. For instance constitutive deletions of ARPC3 or APRC4 are both embryonically lethal, whereas work has progressed only to ES cell development for knockouts of APR2 and APRC5 [127].

Further study of platelet formin proteins is also warranted. Platelets express multiple formins and here, as with the GTPases, there is likely to be redundancy. Targeted inhibition of these proteins potentially in combination with pharmacological inhibition or gene deletion of probable Rho GTPase activators may aid the search. For instance mDia1 is known to be dispensable for platelet filopodia formation [86]. This formin protein binds to both Rif [61], and to Cdc42 via IRSp53 [62]. It would be intriguing to see what effects removal or inhibition of multiple formin proteins for example mDia1 and mDia3 (leaving only mDia2) or combined deletion of Cdc42 and mDia1 (favouring Rif–mDia3) might produce in platelets. The availability of all of these relevant mice lines means that such experiments are within reach.

### What GEFs, GAPs and GDIs regulate platelets?

Signalling via small GTPases is determined by their spatial and temporal distribution and by numerous regulating proteins (Table 2) [128]. Rho GTPases may either be GDP-loaded and inactive or GTP-loaded and active. Since GDP is typically tightly bound and the rate of hydrolysis of GTP is slow, Rho GTPases require the activity of two additional types of protein to function efficiently [129]. Activation of Rho GTPases occurs via the GTP loading activity of GEFs, which catalyse the exchange of bound GDP for GTP.

**Table 2:** A list of the most prevalent Rho GTPase GEFs, GAPs and GDIs in human platelets with their known target GTPases The list was based on Takai et al. [147] augmented with information from domain homology searches conducted using protein sequence (www.uniprot.org/) and protein interaction (string-db.org/) databases. Table entries were cross-referenced against two recent proteomic databases such that only proteins known to be expressed are listed [145,146], and then ranked using mRNA transcript levels from human platelets [35].

UniProt ID	Gene name	Protein name	GTPase target
Q9NZN5	ARHGEF12	Rho guanine nucleotide exchange factor 12	RhoA, RhoB, Rac
Q5JSP0	FGD3	FYVE, RhoGEF and PH domain-containing protein 3	Cdc42
Q92888	ARHGEF1	Rho guanine nucleotide exchange factor 1	RhoA
Q9NR81	ARHGEF3	Rho guanine nucleotide exchange factor 3	RhoA, RhoB, Cdc42
O60229	KALRN	Kalirin	Rac
Q8TCU6	PREX1	P-Rex1 (PtdIns(3,4,5)-dependent Rac exchanger 1)	Rac
Q9UKW4	VAV3	Guanine nucleotide exchange factor VAV3	Rac, Cdc42
O75962	TRIO	Triple functional domain protein (TRIO)	Rac, RhoA
Q15052	ARHGEF6	Rho guanine nucleotide exchange factor 6	Rac, Cdc42
Q8NF50	DOCK8	Dedicator of cytokinesis protein 8	Rac, Cdc42
Q6ZSZ5	ARHGEF18	Rho guanine nucleotide exchange factor 18	RhoB, Rac
Q9H7D0	DOCK5	Dedicator of cytokinesis protein 5	RhoA, Rac
Q92974	ARHGEF2	Rho guanine nucleotide exchange factor 2	RhoA
Q07889	SOS1	Son of sevenless homologue 1 (SOS-1)	Rac
Q92608	DOCK2	Dedicator of cytokinesis protein 2	Rac
P15498	VAV1	Proto-oncogene Vav	Rac, Cdc42
Q9NZM3	ITSN2	Intersectin-2	Cdc42
Q5T5U3	ARHGAP21	Rho GTPase-activating protein 21	RhoA, Cdc42
Q8N392	ARHGAP18	Rho GTPase-activating protein 18	Rac, RhoQ, RhoU
O43182	ARHGAP6	Rho GTPase-activating protein 6	Rac
P11274	BCR	Breakpoint cluster region protein	Rac, Cdc42
Q96P48	ARAP1	Arf-GAP with Rho-GAP, ANK repeat, PH domains 1	?
Q9P107	GMIP	GEM-interacting protein	RhoA
P98171	ARHGAP4	Rho GTPase-activating protein 4	Rac
Q92502	STARD8	StAR-related lipid transfer protein 8	RhoA, Cdc42
Q07960	ARHGAP1	Rho GTPase-activating protein 1	Cdc42
Q7Z6I6	ARHGAP30	Rho GTPase-activating protein 30	Rac, RhoH
Q9BRR9	ARHGAP9	Rho GTPase-activating protein 9	Cdc42, Rac, RhoA
O60890	OPHN1	Oligophrenin-1	RhoA, Rac
A1A4S6	ARHGAP10	Rho GTPase-activating protein 10	RhoA, Cdc42
P42331	ARHGAP25	Rho GTPase-activating protein 25	?
Q12979	ABR	Active breakpoint cluster region-related protein	Rac, Cdc42
Q6ZUM4	ARHGAP27	Rho GTPase-activating protein 27	Cdc42, Rac
Q8N103	TAGAP	T-cell activation Rho GTPase-activating protein	?
P52566	ARHGDIB	Rho GDP-dissociation inhibitor 2	Rac, RhoA, RhoB, RhoH
P52565	ARHGDIA	Rho GDP-dissociation inhibitor 1	Rac, RhoA, RhoH

To facilitate the regulation of the various platelet Rho GTPases, platelets contain a large number of Rho GEFs. Some GEFs, including PIP3-dependent Rac exchanger 1 protein (PREX-1), specifically interact with certain GTPases *in vivo* [130], whereas others such as Vav are more promiscuous and can activate multiple Rho family members. Many GEFs including Vav and RhoGEF12 exist in auto-inhibited conformations. In the case of Vav, activation of the GEF requires phosphorylation of several tyrosine residues to unlock the protein and expose the GEF active site [129]. Such regulation presumably explains why Vav associates with the LAT signalsome of tyrosine kinases assembled following collagen stimulation of GPVI [131].

In contrast, TRIO has not been characterized in platelets to date, although various reagents exist that would enable initial investigation of its expression and regulation. For instance, it has been demonstrated that TRIO is phosphorylated at Tyr2622 by the Src kinase Fyn in response to upstream receptor stimulation in cultured neurons [123]. An inhibitor of TRIO (ITX-3) has also recently been reported and could be used to determine if TRIO activates RhoG in platelets and more generally to investigate the role of TRIO in platelets [132]. Knockout mice with both conditional and constitutive targeted mutations of TRIO have been produced and would also facilitate detailed investigation of the role of this GEF in platelets [133].

Interestingly, in a recent AP-MS study using GST-RhoG, no probable GEF proteins for RhoG were identified [112]. This was likely due to the use of a minimally truncated native RhoG bait protein. Although this protein was suitable for nucleotide exchange and the identification of GTPase activating proteins (GAPs) and effector proteins, it was less suitable for identification of GEFs. Studies that specifically aimed to identify GEF proteins have employed dominant negative mutants with single amino acid substitutions (RhoG G15A, RhoA G17A) [134,135]. These amino acid substitutions cause the protein to bind very poorly to both GDP and GTP [136], making the protein essentially nucleotide-free. This state of Rho GTPase proteins is an intermediate within the nucleotide exchange reaction enabling these mutant proteins to form high affinity binary complexes with GEFs [137,138].

Currently, the roles of GAPs in platelets are poorly characterized [1,139]. Several Rho GAPs in platelets have now been described including p190RhoGAP which acts on RhoA [1], nadrin which acts on Rac and Cdc42 [140], and oligophrenin-1 [141], which has recently been demonstrated to play a role regulating platelet filopodia formation [142]. Use of constitutively active Rho GTPase mutants to preferentially capture GAPs may increase the likelihood of identifying these regulators in platelets.

Four Rho GDIs are expressed in platelets, and proteomics data suggest that RhoGDIα is highly expressed (21700 copies per platelet) and therefore represents a plausible candidate for the inhibitory regulation of Rho GTPases in platelets [38]. Our lack of understanding of GDI function in platelets was recently highlighted, and any data regarding the functions of these proteins in platelets will be novel [1].

### How do Rho GTPases contribute to the pathological roles of platelets?

Rho GTPase regulated platelet secretion is likely to be involved in the roles played by platelets in the development and progression of various disease processes including atherogenesis, asthma and cancer. Few (if any) suitable agents exist for the selective inhibition of Rho GTPases to reduce platelet secretion, however, selective modulation of Rho GTPase activity through inhibition of upstream GEFs may hold more promise [95,132,143]. In addition, investigation of the pathogenesis of inflammatory or neoplastic disease processes within mice lacking specific Rho GTPases may provide insights into the role of platelet secretion in disease and determine if such GEF inhibition holds any promise for future therapeutic interventions.
